# Supplementary material for: Molecular response to PARP1 inhibition in ovarian cancer cells as determined by mass spectrometry based proteomics
Source: J Ovarian Res. 2021 Oct 22;14:140. doi: 10.1186/s13048-021-00886-x (PMC8539835; doi:10.1186/s13048-021-00886-x)
Supplement: Supplementary file 1 — Additional file 1: Supplementary Figure (SFigure) 1. (a) Pearson correlation coefficients (r) were calculated for biological replicates (n = 3) of samples. (b) Quantification of sample comparability determined by coefficient of variation (CV) of replicates. [file 13048_2021_886_MOESM1_ESM.pdf]

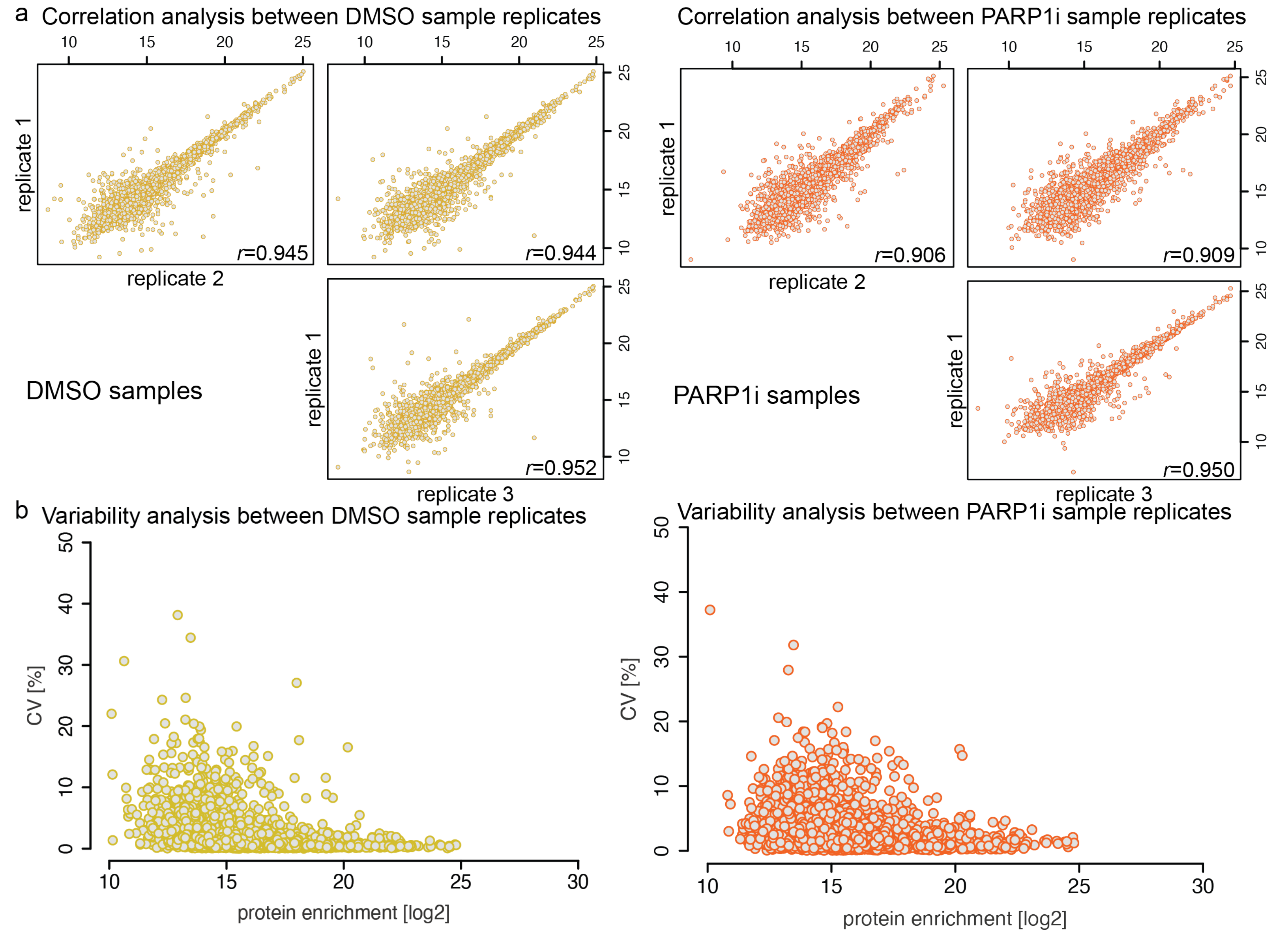
a
Correlation analysis between DMSO sample replicates
Correlation analysis between PARP1i sample replicates
replicate 1
replicate 2
replicate 3
r=0.945
r=0.944
r=0.952
r=0.906
r=0.909
r=0.950
DMSO samples
PARP1i samples
b
Variability analysis between DMSO sample replicates
Variability analysis between PARP1i sample replicates
CV [%]
protein enrichment [log2]
