# Supplementary figures and images for: Molecular response to PARP1 inhibition in ovarian cancer cells as determined by mass spectrometry based proteomics
Source: J Ovarian Res. 2021 Oct 22;14:140. doi: 10.1186/s13048-021-00886-x (PMC8539835; doi:10.1186/s13048-021-00886-x)

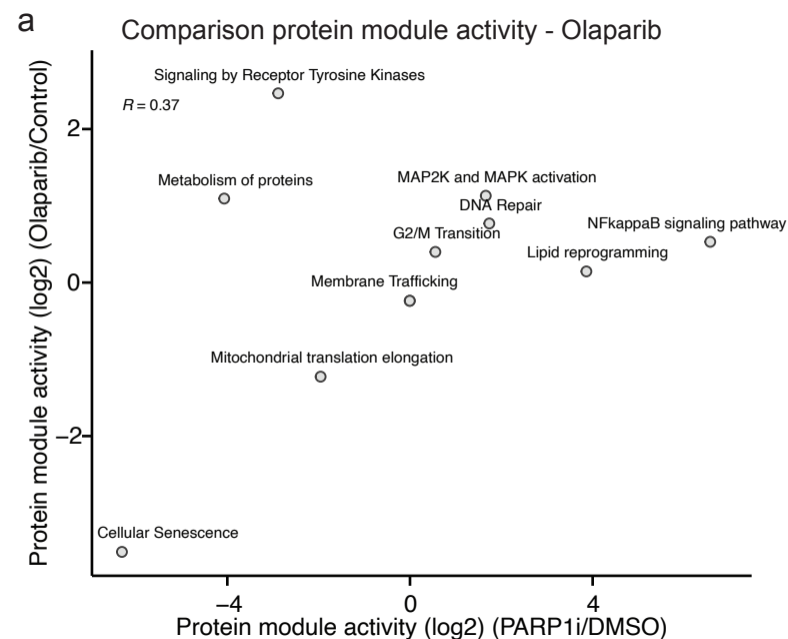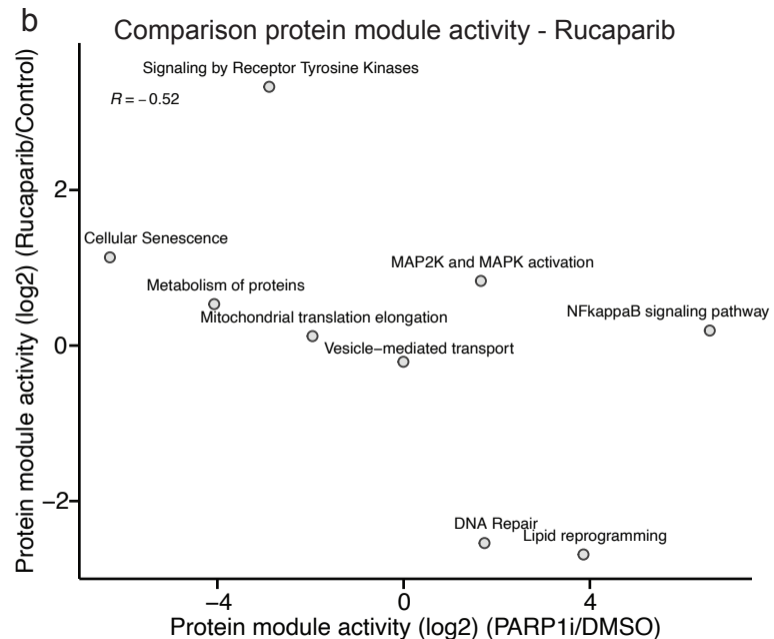

Supplement: Supplementary file 2 — Additional file 2: Supplementary Figure (SFigure) 2. (a, b) Correlation analysis of the overall protein module activity based on overlapping annotated modules between L1000 olaparib-treated cell lines (a) and rucaparib-treated cell lines (b) and proteome profiling. Activity was calculated by integrating protein expression changes in modules [38] (Methods). [file 13048_2021_886_MOESM2_ESM.pdf]
